# Prevalence and impact of *Clostridium difficile* infection in elderly residents of long-term care facilities, 2011: A nationwide study

**DOI:** 10.1097/MD.0000000000004187

**Published:** 2016-08-07

**Authors:** Panayiotis D. Ziakas, Nina Joyce, Ioannis M. Zacharioudakis, Fainareti N. Zervou, Richard W. Besdine, Vincent Mor, Eleftherios Mylonakis

**Affiliations:** a Department of Medicine, Warren Alpert Medical School; b Center for Gerontology and Healthcare Research, Brown University, Providence, RI; c Department of Health Care Policy, Harvard Medical School, Boston, MA; d Infectious Diseases Division, Warren Alpert Medical School, Brown University; e Providence Veterans Administration Medical Center, Center for Innovation (COIN), Providence, RI.

**Keywords:** *C difficile*, epidemiology, long-term care, mortality, nursing home, prevalence, risk factors

## Abstract

The elderly population is particularly vulnerable to *Clostridium difficile* infection (CDI), but the epidemiology of CDI in long-term care facilities (LTCFs) is unknown.

We performed a retrospective cohort study and used US 2011 LTCF resident data from the Minimum Data Set 3.0 linked to Medicare claims. We extracted CDI cases based on International Classification of Diseases-9 coding, and compared residents with the diagnosis of CDI to those who did not have a CDI diagnosis during their LTCF stay. We estimated CDI prevalence rates and calculated 3-month mortality rates.

The study population consisted of 2,190,613 admissions (median age 82 years; interquartile range 76–88; female to male ratio 2:1; >80% whites), 45,500 of whom had a CDI diagnosis. The nationwide CDI prevalence rate was 1.85 per 100 LTCF admissions (95% confidence interval [CI] 1.83–1.87). The CDI rate was lower in the South (1.54%; 95% CI 1.51–1.57) and higher in the Northeast (2.29%; 95% CI 2.25–2.33). Older age, white race, presence of a feeding tube, unhealed pressure ulcers, end-stage renal disease, cirrhosis, bowel incontinence, prior tracheostomy, chemotherapy, and chronic obstructive pulmonary disease were independently related to “high risk” for CDI. Residents with a CDI diagnosis were more likely to be admitted to an acute care hospital (40% vs 31%, *P* < 0.001) and less likely to be discharged to the community (46% vs 54%, *P* < 0.001) than those not reported with CDI during stay. Importantly, CDI was associated with higher mortality (24.7% vs 18.1%, *P* = 0.001).

CDI is common among the elderly residents of LTCFs and is associated with significant increase in 3-month mortality. The prevalence is higher in the Northeast and risk stratification can be used in CDI prevention policies.

## Abstract 2

Supplemental Digital Content is available in the text

## 1 Introduction

*Clostridium difficile* is the most common cause of acute infectious diarrhea in the hospital setting as well as in long-term care facilities (LTCFs),^[1]^ and disproportionately affects individuals who are >65 years old.^[2]^ Although the incidence of other healthcare-associated infections has declined, the incidence of *C difficile* infections (CDIs) has increased and is the most common hospital infection, representing 12.1% of healthcare-related infections in 2011.^[3]^ The burden and medical care costs of CDIs have reached historic heights and the estimated number of deaths attributed to CDI, based on multiple cause-of-death mortality data, increased from 3000 deaths per year in 1999 to 2000 to 14,000 in 2006 to 2007 with >90% of deaths among persons aged ≥65 years.^[4]^ In 2009, the annual economic burden of CDI in the United States was $8.2 billion,^[5]^ or 2.3% of all hospital costs. These figures also seem to have increased, with other estimations ranging up to $3.2 billion.^[6–8]^ As a result, the Centers for Disease Control and Prevention has categorized *C difficile* as 1 of the 3 microorganisms with a “Threat Level of Urgent.”^[9]^

LTCF residents represent a subset of elderly people particularly vulnerable to CDI. Environmental factors, such as residence in close, shared quarters, shared toilet facilities, and limited ability to isolate infected residents, as well as the intrinsic characteristics of this population, such as the advanced age, immune and physiologic senescence, and multiple comorbid conditions, all contribute to their increased susceptibility to CDI.^[10]^ As data on the epidemiology and risk factors of CDI among elderly residents are limited, we drew on data from Minimum Data Set (MDS) 3.0, the federally mandated nursing home resident assessment questionnaire, linked to Medicare claims to describe the prevalence and correlates of CDI in LTCF residents admitted in 2011.

## 2 Methods

We used data from the MDS 3.0 linked to Medicare claims to study the epidemiology of *C difficile* in 2011 among LTCF residents >65 years old. MDS is a federally mandated resident assessment tool (available at: http://www.cms.gov/Medicare/Quality-Initiatives-Patient-Assessment-Instruments/NursingHomeQualityInits/index.html).^[11–13]^ Resident data (including demographics, diagnosis, and functioning) are recorded on admission and at least quarterly thereafter by LTCF nurses, with high interobserver reliability.^[14]^ The MDS data are available in a national repository, which we accessed through a data use agreement with the Centers for Medicare & Medicaid Services (DUA #28056) for this project. The Institutional Review Board approved the study (Brown University IRB #1410001151).

We summarized the characteristics of the study population using descriptive methods of data analysis. Resident data were retrieved from the pertinent MDS 3.0 sections to include demographics (Section A), active diagnoses (Section I), special treatments and procedures (Section O), swallowing/nutritional status (Section K), bladder and bowel (Section H), and skin conditions (Section M). We a priori sought to retrieve the following specific information: patient demographics (age, sex, race, length of LTCF stay) and medical comorbidities present (including diabetes mellitus, hypertension, chronic obstructive pulmonary disease [COPD], coronary artery disease, Parkinson disease, stroke, dementia, cirrhosis, end-stage renal disease [ESRD], and prior exposure to chemotherapy and/or irradiation). We also included the presence of a feeding tube, bowel/urine incontinence, prior tracheostomy, and the presence of unhealed pressure ulcers as comorbidities that reflect the residents’ performance status. On MDS assessments, CDI reporting relies on the healthcare practitioner to mark the condition as “additional active diagnosis” and there is no checkbox for the exclusive documentation of CDI. CDI diagnosis was based on the International Classification of Diseases (ICD)-9-CM code for “an intestinal infection with *C difficile*” (008.45). Each additional diagnosis (if present) is coded by LTCF personnel in the appropriate box according to ICD-9 classification. We extracted the specific code from the pertinent box, as originally coded, to ascertain CDI.

Comparison of residents with and without CDI during their LTCF stay was performed using the χ^2^ test for categorical variables and the Wilcoxon rank-sum test for continuous data. We performed logistic regression modeling to adjust for significant confounders associated with CDI. We further addressed the impact of CDI on 3-month mortality (defined as death from any cause within the next 90 days after first CDI assessment), using Medicare enrollment data.

CDI prevalence was defined as the proportion of CDI cases per LTCF admissions in 2011. Stratified CDI rates were presented by state and by geographic region (Northeast, Midwest, South, West) according to US Census Bureau grouping and definitions in the National Nursing Home Survey.^[15]^ Stata v.14 (College Station, TX) was used for data analysis. The study is STROBE-compliant and provides the pertinent checklist as per journal requirements.

## 3 Results

**Figure 1:**
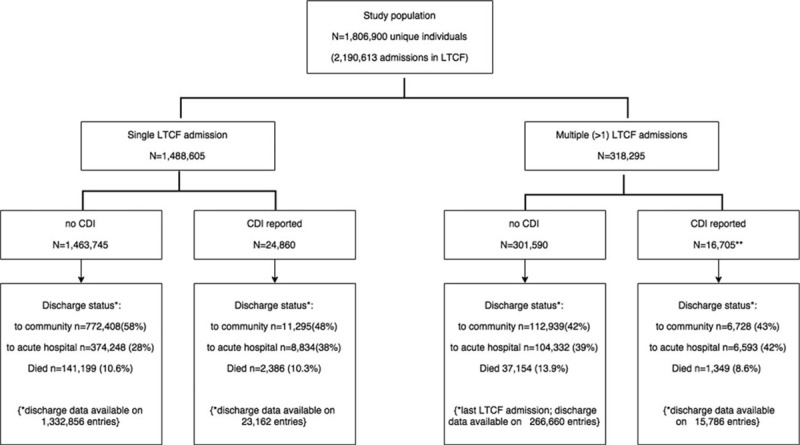
Profile of the present study. CDI = *C difficile* infection, LTCF = long-term care facility.

Overall, 2,190,613 admissions (1,806,900 unique individuals) aged 65 or older were included in the analysis. Specifically 1,488,605 individuals had a single admission in 2011, whereas 318,295 individuals had >1 LTCF admission during 2011, totaling 702,008 admissions (average 2.2 admissions for individuals with multiple admissions). The study profile is illustrated in Fig. 1. Their median age was 82 years (interquartile range 76–88) and females outnumbered male residents (approximately 2:1 ratio). Approximately 82% were white, followed by African American (9%), Hispanic (4%), and mixed or other origin (2%). Comorbidities included, in descending order, hypertension (69%), diabetes mellitus (29%), dementia (26%), coronary artery disease (24%), COPD (21%), ESRD (13%), stroke (12%), Parkinson disease (4%), and cirrhosis (0.5%). The median length of resident stay was 33 days (interquartile range, 19–90 days). Approximately 16% had unhealed pressure ulcers, 18% had urinary incontinence, 10% had bowel incontinence, and 4% had a feeding tube. Less than 1% had prior tracheostomy or received chemotherapy or irradiation (Table 1).

**Table 1:**
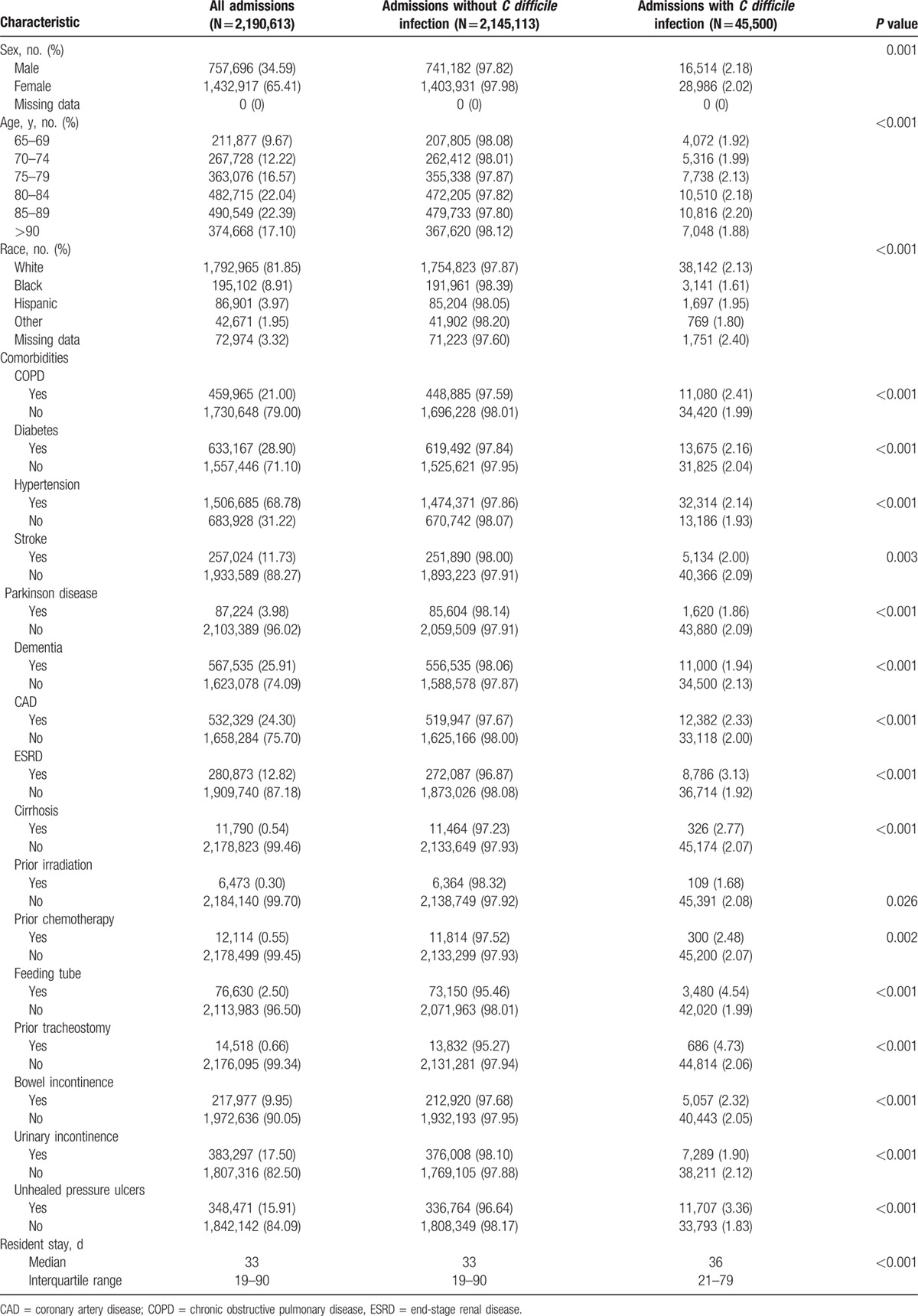
Demographics and clinical characteristics of surveyed residents.

CDI was reported in 45,500 of admissions during LTCF stay (2.08%), representing 41,565 unique individuals. The stratified data (by demographics and comorbidities) are summarized in Table 1. The rate was higher among whites (2.13%) and lowest among African Americans (1.61%). Across medical comorbidities, the highest rates were noted among residents with tracheostomy (4.73%), feeding tube (4.54%), unhealed pressure ulcers (3.36%), ESRD (3.13%), and cirrhosis (2.77%). CDI varied across different age strata, being higher (2.20%) for the 85 to 89 age group.

**Figure 2:**
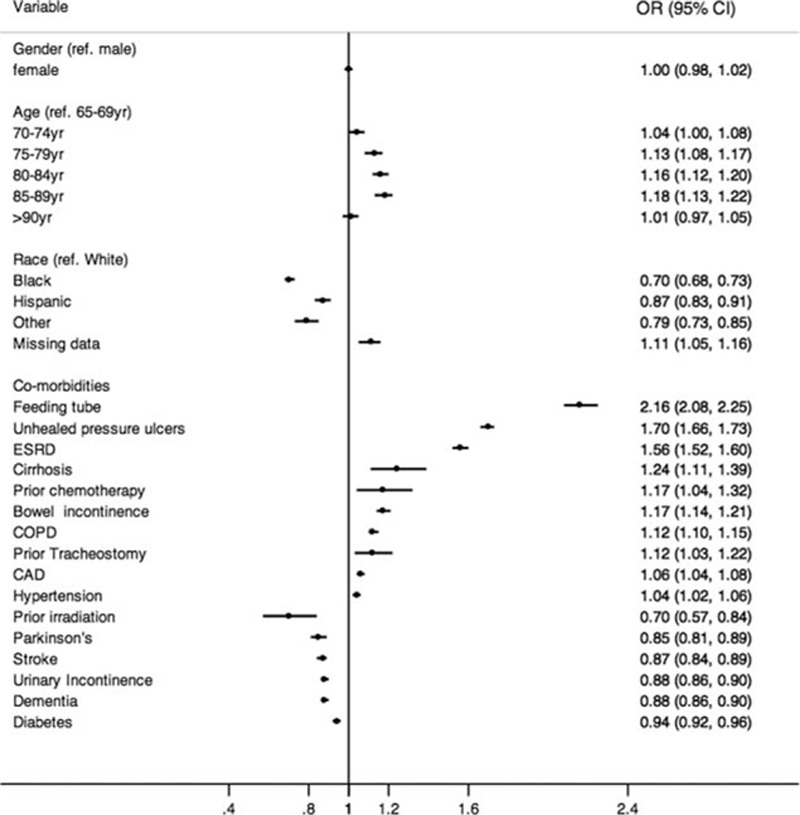
Multivariable analysis. CDI is the dependent outcome. CDI = *C difficile* infection, CI = confidence interval, COPD = chronic obstructive pulmonary disease, ESRD = end-stage renal disease, OR = odds ratio.

In multivariable analysis for risk stratification (full model in Table S1 in Supplementary Appendix), the CDI risk was lower among nonwhite populations and increased with age. No sex differences were noted. Across comorbidities, the presence of a feeding tube, unhealed pressure ulcers, ESRD, cirrhosis, prior chemotherapy, bowel incontinence, prior tracheostomy, and COPD were influential factors associated with CDI (Fig. 2).

**Figure 3:**
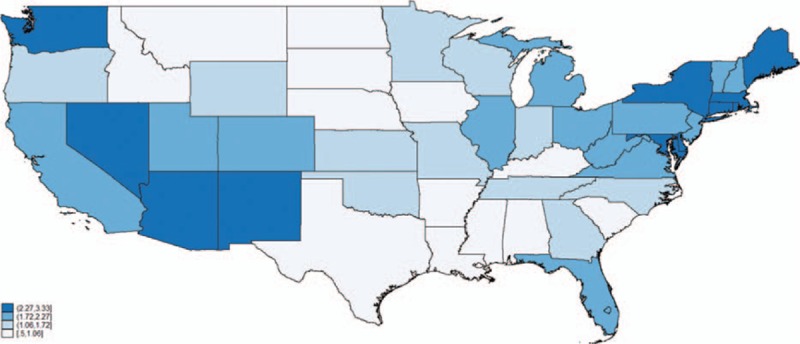
US choropleth map illustrating CDI rates by state (quartile distribution of point estimates). Template state map derived as Environmental Systems Research Institute shapefile from the National Weather Service (available at: http://www.nws.noaa.gov/geodata/catalog/national/html/us_state.htm). Hawaii and Alaska territories (not seen in the map) belong to the lowest quartile. CDI = *C difficile* infection.

The nationwide 2011 prevalence rates, stratified by state, appear in Table S2, and are graphically illustrated in Fig. 3. The crude CDI rate was 1.85% (95% confidence interval [CI] 1.83–1.87) and the rate was higher in the Northeast (2.29%; 95% CI 2.25–2.33) and lower in the South (1.54%; 95% CI 1.51–1.57) (Table 2). Overall, 26,268 of 41,565 individuals (63%) with CDI in LTCF had recent hospitalization (within the prior 30 days) and/or had been discharged with CDI from the hospital within the previous 90 days, which leaves a total of 37% CDI cases that are not clearly related to prior healthcare exposure and may represent “true” LTCF-related cases. CDI was reported more than once in 3935 of 16,705 (24%) individuals who had >1 LTCF admission in 2011.

**Table 2:**
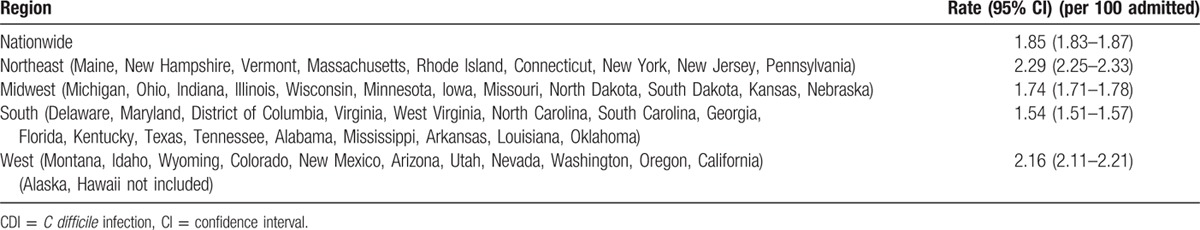
CDI prevalence (%) rates in long-term care facilities grouped by region (as used by the US Census Bureau).

Table 3 displays the cumulative discharge data. LTCF residents with a CDI diagnosis were more likely to be admitted to an acute care hospital (40% vs 31%, *P* < 0.001) and less likely to be discharged to the community (46% vs 54%, *P* < 0.001) than those not reported with CDI during stay. The crude LTCF mortality rates were 9.0% and 8.7% (*P* = 0.01), respectively.

**Table 3:**
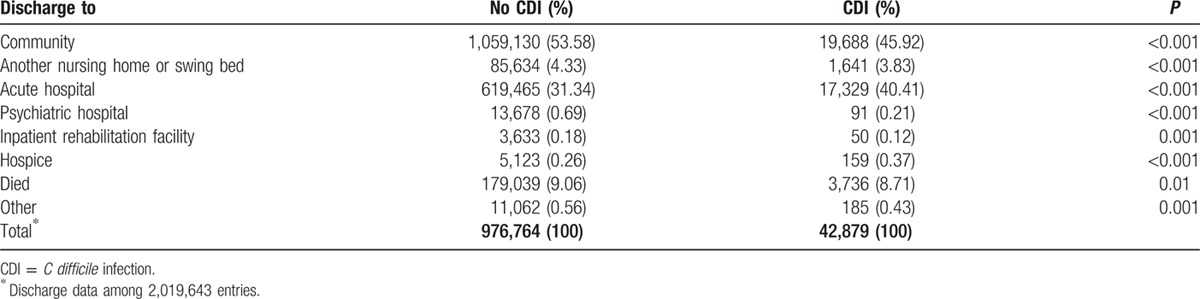
Outcome of long-term care facility stay.

**Figure 4:**
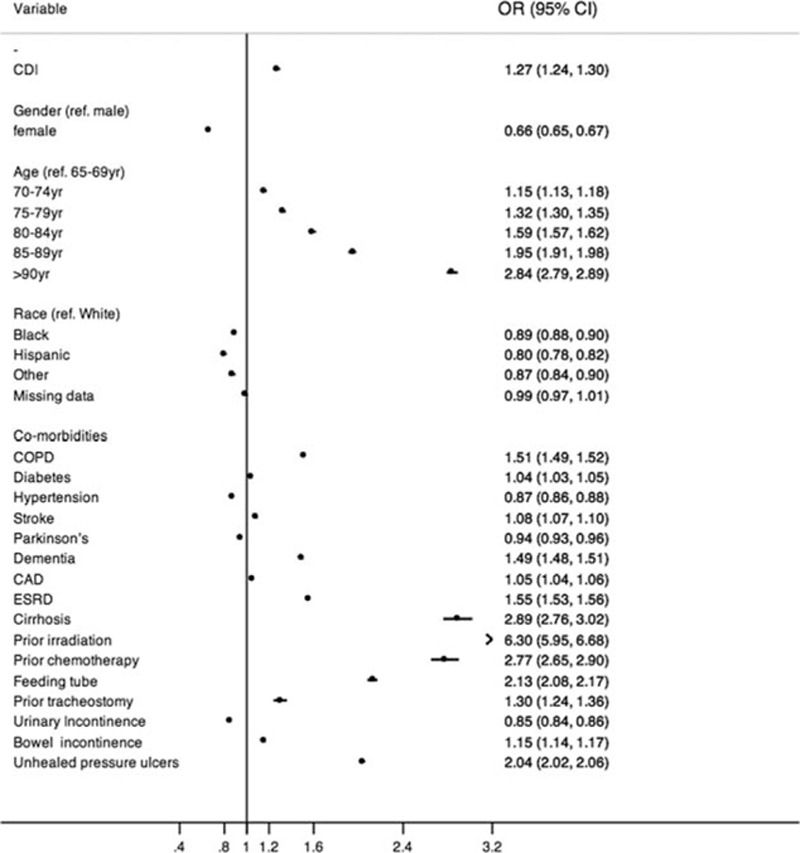
Multivariable analysis. The 3-month mortality is the dependent outcome. CDI = *C difficile* infection, CI = confidence interval, COPD = chronic obstructive pulmonary disease, ESRD = end-stage renal disease, OR = odds ratio.

Survival data were available for 1,754,553/1,806,900 (97%) of individuals included in the study. The 3-month mortality rates were 24.7% (8924/36,164) for residents with a CDI diagnosis at LTCF compared to 18.1% (310,358/1,718,389) for those without CDI (*P* < 0.001). Importantly, CDI was independently associated with mortality (Fig. 4), in multivariable analysis (full model in Table S3 in Supplementary Appendix), with the odds being 27% higher for those with reported CDI (adjusted odds ratio 1.27, 95% CI 1.24–1.30).

## 4 Discussion

Our results indicate that approximately 1 out of every 50 residents aged 65 or older admitted in a Medicare-certified LTCFs was diagnosed with CDI during his or her stay. The nationwide CDI estimate in this setting is reported for the first time and the presence of geographic differences across the United States is depicted. Importantly, more than one-third of CDI cases appear to be independent of recent hospitalization to an acute care facility. CDI recorded at LTCF was independently associated with specific risk factors, and is correlated with a higher overall mortality.

Hospital discharge data are suggestive of an increasing proportion of patients with CDI^[16]^ and more than half of LTCF admissions derive from acute care hospitals and hospital-based facilities.^[15]^ Taken together these findings outline the need to evaluate the burden of CDI in LTCFs. Defining the origin of CDI is important, because imported cases will reflect poor prevention policies and antibiotic misuse in the hospital setting, rather than in LTCF. Indeed, we showed that almost two-thirds of CDI cases in LTCFs had been previously discharged with CDI from hospital or had recent hospitalization in an acute care facility, which underscores the fact that the majority of CDI that develop in the LTCF setting may “originate” in the acute hospital setting.^[17]^ However, LTCF environment should be regarded as a major component of CDI epidemics as more than one-third of CDI cases may be regarded as “true” LTCF-related cases.

On the other hand, the distinction of LTCF-associated cases may reflect transmission patterns and inadequacy of infection control in the LTCF setting. Transmission in the LTCFs likely occurs by direct spread from the hands of personnel, fomites, and LTCF environment^[18–20]^ and is facilitated by the fact that residents live in shared quarters, including sleeping, eating, and toileting facilities, and that the assignment of patients to private rooms does not seem to be feasible in most cases.^[21]^ The fact that management of CDI is challenging in these facilities^[10,22–24]^ highlights the importance of primary prevention of CDI in this patient population.

Identifying factors that are associated with increased risk of CDI may indicate a subpopulation for future preventive strategies. To date, limited data exist regarding risk factors for CDI in LTCF and the pertinent information was derived from a number of studies conducted in the hospital setting.^[10]^ The presence of comorbid conditions,^[25]^ the prolonged length of stay,^[26]^ the use of feeding tubes,^[25]^ overexposure to acid-suppressant medications,^[26]^ and frailty^[27,28]^ have been sporadically implicated in CDI epidemiology in LTCF. We found that indicators of more debilitated individuals and specific comorbidities including cirrhosis and ESRD may also contribute to CDI susceptibility. Plausible explanations are the frequent hospitalizations and interaction of these patients with the hospital environment (e.g., patients with ESRD), the use of broad-spectrum antibiotics for treatment of infections (e.g., decubitus ulcers) and for prophylaxis (e.g., for patients with cirrhosis), and the bypassing of the gastric barrier through nasogastric/enteral feeding or the increased use of antacid medications.^[29–33]^ Of note is the fact that the association of fecal incontinence with increased CDI rates may be partially inflated by more frequent CDI testing in this subgroup of individuals.^[34]^

CDI is a worldwide epidemic with worrisome expansion, and a growing challenge for health experts. It is not confined to LTCFs in USA but also extends in several geographic areas overseas, affecting the elderly and LTCF setting.^[35–38]^ The adoption of prevention policies across the continuum of care is necessary in order to control the epidemic, but it is unlikely that antibiotic stewardship programs^[39,40]^ and infection control policies will suffice to solve the problem across the healthcare system.^[10,22]^ Moreover, bacteria can continue to adapt.^[41,42]^ In a bigger picture, the profile related to CDI in the elderly LTCF population documented in the present study unfolds the adverse impact of chronic disease and increased contact with the healthcare system on CDI epidemic. Inevitably, prevention policies should expand beyond the static viewpoint of disease management, aiming to promoting healthy living. Therefore, interventions that promote healthy living or alter environmental influences before disease develops can be more effective in controlling chronic illness^[43]^ and limiting healthcare exposure. For example, COPD can be prevented by successful campaigns favoring tobacco cessation, and cirrhosis by alcohol restriction legislation; obesity and related disorders such as coronary artery disease and hypertension may require calorie restriction policies including fiscal measures as a public health tool (using the example of Mexico on imposing tax on sweetened beverages).^[44,45]^ Living in healthy cities^[46]^ that adopt evidence-informed health strategies and put health high in their social, political, and environmental priorities may also contribute to disease control, as urbanization and socioeconomic status differences impact population health.^[47–49]^ CDI epidemic in the LTCF is a paradigm that justifies a shift from unidimensional, disease-based prevention policies to multidimensional and dynamic healthcare.^[50]^

### 4.1 Limitations

Analysis of official registries does not provide detailed individual data, but the large sample size and the nationwide coverage add to the importance and validity of the present study. Moreover, socioeconomic status, such as income, education level, and social status that may influence disease profile and outcome were not available in the present study. Also, pertinent data on antimicrobial drug use, proton pump inhibitors/histamine H_2_-receptor antagonists, and individual data such as body mass index or obesity were also not available.

Our definition of CDI was based on the ICD-9-CM code for “an intestinal infection with *C difficile*,” whose sensitivity and specificity are largely unknown, with previous studies showing that it may underestimate or overestimate the true burden of the disease.^[51,52]^ However, in the absence of a national surveillance system for CDI, ICD-9 code provides an easily accessible tool for estimating the burden of this infection.^[51]^ Moreover, for the estimation of CDI episodes during the LTCF stay we used the MDS v3.0 forms, in which there is no checkbox for the documentation of CDI exclusively. On MDS assessments, the identification of CDI relies on the healthcare practitioner to input the diagnosis, as opposed to check a box. This may result in an underreporting of CDI in LTCFs and may induce selection bias in the form of selecting patients with the most severe clinical characteristics. As such, our estimations probably underestimate the actual burden of CDI. Along with the potential insensitivity of coding, limitations involve the different methods used for isolation of *C difficile* in different LTCFs and hospitals all over the United States. Furthermore, the analysis data are representative only of patients in Medicare fee-for-service and do not reflect the population enrolled in Medicare-managed care, for whom insurance claims are not collected. The data set does not contain information regarding services not covered by, or billed to, Medicare and how that might affect the results. Finally the ratio of “true” LTCF-related cases is likely overestimated, because a number of CDI cases categorized as such may have acquired *C difficile* in the community and become symptomatic in LTCF. Additionally, the analysis of MDS form does not permit the distinction between primary CDI episode and CDI recurrences, which might significantly affect estimates on LTCF-acquired CDI. Such analysis would require longitudinal follow-up on a per patient basis that should also cover the community setting.

## 5 Conclusions

Our study estimated the nationwide burden of CDI infection among the elderly population in LTCF and provided the rationale for targeted prevention policies. Identification of LTCF residents who are at high risk for CDI may provide insights into interventions for future prevention strategies studied exclusively in this patient population. These efforts should focus on LTCF residents who are at high risk, such as those having a feeding tube or pressure ulcers, and those with ESRD and cirrhosis. Importantly, we report that both CDI in the LTCF and the hospital setting appear to feed the CDI epidemic, as up to two-thirds of CDI cases may be related to hospitalization in acute care facilities, but a significant proportion of CDI is related to LTCF. Further research could benefit from the addition of a separate checkbox in MDS form to uniquely code for CDI, allowing opting for primary infection or recurrences. Acute care facilities and LTCFs should collaborate in reducing CDI rates across the continuum of care.

## Supplementary Material

Supplemental Digital Content
