# Supplementary material for: Prevalence and impact of Clostridium difficile infection in elderly residents of long-term care facilities, 2011: A nationwide study
Source: Medicine (Baltimore). 2016 Aug 7;95(31):e4187. doi: 10.1097/MD.0000000000004187 (PMC4979776; doi:10.1097/MD.0000000000004187)
Supplement: Supplemental Digital Content [file medi-95-e4187-s001.doc]

**Table S1: Logistic regression, multi-variable analysis for CDI as the dependent outcome.**

| **Variable** | **Effect size** | **Odds Ratio(95%CI)** | **p value** |
| --- | --- | --- | --- |
| female gender | 0 | 1.00 (0.98-1.02) | 0.96 |
| Age-no |  |  |  |
| 65-69yr | ref | Ref |  |
| 70-74yr | .040 | 1.04(1.00-1.08) | 0.05 |
| 75-79yr | 0.118 | 1.13(1.08-1.17) | <0.001 |
| 80-84yr | 0.147 | 1.16(1.12-1.20) | <0.001 |
| 85-89yr | 0.163 | 1.18(1.13-1.22) | <0.001 |
| >90yr | 0.006 | 1.01(0.97-1.05) | 0.78 |
| Race |  |  |  |
| White | Ref | Ref |  |
| Black | -0.355 | 0.70(0.68-0.73) | <0.001 |
| Hispanic | -0.140 | 0.87(0.83-0.91) | <0.001 |
| Other | -0.239 | 0.79(0.73-0.85) | <0.001 |
| Missing data | 0.101 | 1.11(1.05-1.16) | <0.001 |
| Co-morbidities |  |  |  |
| Feeding tube | 0.773 | 2.16(2.08-2.25) | <0.001 |
| Unhealed pressure ulcers | 0.529 | 1.70(1.66-1.73) | <0.001 |
| ESRD | 0.444 | 1.56(1.52-1.60) | <0.001 |
| Cirrhosis | 0.216 | 1.24(1.11-1.39) | <0.001 |
| Prior chemotherapy | 0.161 | 1.17(1.04-1.32) | 0.007 |
| Bowel incontinence | 0.158 | 1.17(1.14-1.21) | <0.001 |
| COPD | 0.119 | 1.12(1.10-1.15) | <0.001 |
| Prior Tracheostomy | 0.111 | 1.12(1.03-1.22) | 0.01 |
| CAD | 0.057 | 1.06(1.04-1.08) | <0.001 |
| Hypertension | 0.038 | 1.04(1.02-1.06) | <0.001 |
| Prior irradiation | -0.363 | 0.70(0.57-0.84) | <0.001 |
| Parkinson’s | -0.164 | 0.85(0.81-0.89) | <0.001 |
| Stroke | -0.143 | 0.87(0.84-0.89) | <0.001 |
| Urinary Incontinence | -0.128 | 0.88(0.86-0.90) | <0.001 |
| Dementia | -0.126 | 0.88(0.86-0.90) | <0.001 |
| Diabetes | -0.063 | 0.94(0.92-0.96) | <0.001 |

**Table S2**: Study population and *C.difficile* prevalence rates by State (2011)

| **State** | **N** | **n** | **CDI rate(%)** | **95%CI** |
| --- | --- | --- | --- | --- |
| AK & HI | 6,435 | 32 | 0.50 | 0.32-0.67 |
| AL | 32,508 | 239 | 0.74 | 0.64-0.83 |
| AR | 21,187 | 160 | 0.76 | 0.64-0.87 |
| AZ | 30,982 | 934 | 3.01 | 2.82-3.21 |
| CA | 179,271 | 3,801 | 2.12 | 2.05-2.19 |
| CO | 27,969 | 519 | 1.86 | 1.70-2.01 |
| CT | 41,706 | 1,059 | 2.54 | 2.39-2.69 |
| DC | 2,559 | 40 | 1.56 | 1.08-2.04 |
| DE | 7,225 | 173 | 2.39 | 2.04-2.75 |
| FL | 156,012 | 3,360 | 2.15 | 2.08-2.22 |
| GA | 40,457 | 509 | 1.26 | 1.15-1.37 |
| IA | 27,309 | 271 | 0.99 | 0.87-1.11 |
| ID | 7,700 | 76 | 0.99 | 0.77-1.21 |
| IL | 107,318 | 2,228 | 2.08 | 1.99-2.16 |
| IN | 56,495 | 854 | 1.51 | 1.41-1.61 |
| KS | 22,636 | 286 | 1.27 | 1.11-1.41 |
| KY | 31,527 | 333 | 1.06 | 0.94-1.17 |
| LA | 24,057 | 177 | 0.74 | 0.63-0.84 |
| MA | 67,787 | 1,786 | 2.63 | 2.51-2.76 |
| MD | 42,903 | 1,301 | 3.03 | 2.87-3.19 |
| ME | 13,405 | 311 | 2.32 | 2.07-2.57 |
| MI | 75,622 | 1,571 | 2.08 | 1.98-2.18 |
| MN | 52,135 | 864 | 1.66 | 1.55-1.77 |
| MO | 52,114 | 718 | 1.38 | 1.28-1.48 |
| MS | 18,780 | 94 | 0.50 | 0.40-0.60 |
| MT | 6,199 | 52 | 0.84 | 0.61-1.07 |
| NC | 60,227 | 737 | 1.22 | 1.14-1.31 |
| ND | 6,019 | 54 | 0.90 | 0.66-1.14 |
| NE | 17,633 | 134 | 0.76 | 0.63-0.89 |
| NH | 9,773 | 208 | 2.13 | 1.84-2.41 |
| NJ | 83,589 | 1,890 | 2.26 | 2.16-2.36 |
| NM | 9,668 | 318 | 3.29 | 2.93-3.64 |
| NV | 10,463 | 348 | 3.33 | 2.98-3.67 |
| NY | 150,121 | 3,525 | 2.35 | 2.27-2.42 |
| OH | 123,434 | 2,453 | 1.99 | 1.90-2.07 |
| OK | 22,832 | 315 | 1.38 | 1.23-1.53 |
| OR | 19,109 | 213 | 1.11 | 0.97-1.26 |
| PA | 127,770 | 2,416 | 1.89 | 1.82-1.96 |
| RI | 12,309 | 392 | 3.19 | 2.88-3.49 |
| SC | 26,403 | 224 | 0.85 | 0.74-0.96 |
| SD | 6,670 | 49 | 0.73 | 0.53-0.93 |
| TN | 45,337 | 616 | 1.35 | 1.25-1.46 |
| TX | 132,257 | 1,398 | 1.06 | 1.00-1.11 |
| UT | 12,793 | 228 | 1.78 | 1.55-2.01 |
| VA | 51,059 | 1,159 | 2.27 | 2.14-2.40 |
| VT | 4,596 | 104 | 2.26 | 1.83-2.69 |
| WA | 40,828 | 1,001 | 2.45 | 2.30-2.60 |
| WI | 47,520 | 680 | 1.43 | 1.32-1.54 |
| WV | 13,124 | 236 | 1.80 | 1.57-2.03 |
| WY | 2,640 | 32 | 1.21 | 0.79-1.63 |
|  |  |  |  |  |

N=population size, n= C.difficile Infections recorded, CI=Confidence Interval

**Table S3: Logistic regression, multivariable analysis for 3-month mortality (within 90 days of index CDI) as the dependent outcome. CDI was an independent influential covariate.**

| **Variable** | **Effect size** | **Odds Ratio(95%CI)** | **p value** |
| --- | --- | --- | --- |
| **CDI** | 0.238 | **1.27(1.24-1.30)** | **<0.001** |
| female gender | -0.408 | 0.66(0.65-0.67) | <0.001 |
| Age-no |  |  |  |
| 65-69yr | ref | Ref |  |
| 70-74yr | 0.143 | 1.15(1.13-1.18) | <0.001 |
| 75-79yr | 0.281 | 1.32(1.30-1.35) | <0.001 |
| 80-84yr | 0.466 | 1.59(1.57-1.62) | <0.001 |
| 85-89yr | 0.667 | 1.95(1.91-1.98) | <0.001 |
| >90yr | 1.045 | 2.84(2.79-2.89) | <0.001 |
| Race |  |  |  |
| White | Ref | Ref |  |
| Black | -0.116 | 0.89(0.88-0.90) | <0.001 |
| Hispanic | -0.223 | 0.80(0.78-0.82) | <0.001 |
| Other | -0.141 | 0.87(0.84-0.90) | <0.001 |
| Missing data | -0.013 | 0.99(0.97-1.01) | 0.27 |
| Co-morbidities |  |  |  |
| COPD | 0.411 | 1.51(1.49-1.52) | <0.001 |
| Diabetes | 0.042 | 1.04(1.03-1.05) | <0.001 |
| Hypertension | -0.142 | 0.87(0.86-0.88) | <0.001 |
| Stroke | 0.081 | 1.08(1.07-1.10) | <0.001 |
| Parkinson’s | -0.057 | 0.94(0.93-0.96) | <0.001 |
| Dementia | 0.401 | 1.49(1.48-1.51) | <0.001 |
| CAD | 0.050 | 1.05(1.04-1.06) | <0.001 |
| ESRD | 0.437 | 1.55(1.53-1.56) | <0.001 |
| Cirrhosis | 1.061 | 2.89(2.76-3.02) | <0.001 |
| Prior irradiation | 1.841 | 6.30(5.95-6.68) | <0.001 |
| Prior chemotherapy | 1.020 | 2.77(2.65-2.90) | <0.001 |
| Feeding tube | 0.754 | 2.13(2.08-2.17) | <0.001 |
| Prior tracheostomy | 0.263 | 1.30(1.24-1.36) | <0.001 |
| Urinary Incontinence | -0.162 | 0.85(0.84-0.86) | <0.001 |
| Bowel incontinence | 0.140 | 1.15(1.14-1.17) | <0.001 |
| Unhealed pressure ulcers | 0.714 | 2.04(2.02-2.06) | <0.001 |
